# InterAKTions with FKBPs - Mutational and Pharmacological Exploration

**DOI:** 10.1371/journal.pone.0057508

**Published:** 2013-02-28

**Authors:** Anne-Katrin Fabian, Andreas März, Sonja Neimanis, Ricardo M. Biondi, Christian Kozany, Felix Hausch

**Affiliations:** 1 Research Group Chemical Genomics, Max Planck Institute of Psychiatry, Munich, Germany; 2 Research Group PhosphoSites, Universitaetsklinikum Frankfurt, Medizinische Klinik I, Frankfurt/Main, Germany; University of North Dakota, United States of America

## Abstract

The FK506-binding protein 51 (FKBP51) is an Hsp90-associated co-chaperone which regulates steroid receptors and kinases. In pancreatic cancer cell lines, FKBP51 was shown to recruit the phosphatase PHLPP to facilitate dephosphorylation of the kinase Akt, which was associated with reduced chemoresistance. Here we show that in addition to FKBP51 several other members of the FKBP family bind directly to Akt. FKBP51 can also form complexes with other AGC kinases and mapping studies revealed that FKBP51 interacts with Akt via multiple domains independent of their activation or phosphorylation status. The FKBP51-Akt1 interaction was not affected by FK506 analogs or Akt active site inhibitors, but was abolished by the allosteric Akt inhibitor VIII. None of the FKBP51 inhibitors affected AktS473 phosphorylation or downstream targets of Akt. In summary, we show that FKBP51 binds to Akt directly as well as via Hsp90. The FKBP51-Akt interaction is sensitive to the conformation of Akt1, but does not depend on the FK506-binding pocket of FKBP51. Therefore, FKBP inhibitors are unlikely to inhibit the Akt-FKBP-PHLPP network.

## Introduction

Akt (also called PKB) is a member of the serine-threonine kinase AGC superfamily and has three isoforms (Akt1, Akt2, Akt3). It constitutes an important node in diverse signaling cascades and plays an essential role in cell survival, growth, migration, proliferation, polarity, metabolism, and cell cycle progression [1]. At the physiological level, Akt controls muscle and cardiomyocyte contractility as well as angiogenesis. Because Akt plays a crucial role in the phosphoinositide-3-kinase (PI3K) pathway, which is frequently dysregulated in a wide variety of cancers [2], [3], Akt is a major target for cancer therapy [4]. The Akt inhibitor perifosine is currently evaluated in phase III clinical trials against various cancers whereas the allosteric Akt inhibitor MK-2206 has reached phase I. To overcome the problem of feedback regulation within the PI3K/Akt pathway dual PI3K/mTOR inhibitors seem to be promising and several companies pursue such compounds in phase I or phase II clinical trialsCourtney et al. [5].

Akt is activated by binding of its N-terminal pleckstrin homology (PH) domain to phosphatidylinositol 3,4,5-triphosphate (PIP3), which affects the structure of Akt and recruits it to the plasma membrane. Here, PDK1 phosphorylates the activation loop (T308) and thereby activates Akt [6]. In addition, phosphorylation of the hydrophobic motif (HM) at S473 by mTORC2 [7] is a crucial step for maximal activation of Akt [8].Constitutive phosphorylation on T450 occurs during translation and is required for Akt stability [9]. Protein phosphatase PP2A has been shown to dephosphorylate T308 and thereby inactivate Akt [10]-[11], whereas PHLPP (PH domain leucine-rich repeat protein phosphatase) is a phosphatase known to inactivate Akt by dephosphorylation of S473 [12].

The hydrophobic motif (HM) is characteristic for most AGC kinase family members, including serum- and glucocorticoid-inducible kinase (SGK) and p70 ribosomal S6 kinase (S6K) [13]. The chaperone Hsp90 was shown to maintain stability of SGK and Akt as well as several other kinases by direct interaction with the kinase [14]-[15].The function of Hsp90 is fine-tuned by several accessory cochaperones, including FKBP51 and FKBP52 [16]. They belong to the family of FK506-binding proteins (FKBPs), which display peptidyl-prolyl-cis-trans isomerase (PPIase) activity [17]-[18] In humans, at least 15 FKBPs have been identified [19]. The prototypical FKBP12 consists of only one FK506-binding domain (FK1), which also displays the peptidyl-prolyl-cis-trans isomerase activity. In complex with FKBPs, FK506 or rapamycin induce inhibitory, ternary complexes with calcineurin and mTOR, respectively [20]. FKBP51 consists of the N-terminal FK506-binding domain (FK1) and an additional FKBP-like domain (FK2) with high structural but modest sequence homology to the FK1 domainSchmidt et al. [21]. However, the FK2 domain has neither PPIase activity nor binding affinity to immunosuppressants. At the C-terminus, FKBP51 harbors a tetratricopeptide repeat domain (TPR), where the Hsp90 interaction occurs [22].

Recently, FKBP51 was shown to act as a scaffold protein for the phosphatase PHLPP, thereby negatively regulating the kinase Akt [23]. In a pancreatic cancer xenograft model the positive correlation between the expression of FKBP51 and the response to chemotherapeutics was confirmed *in vivo* [24]. However, diverging results have been reported from several other tumor tissues [25]. Nevertheless, the enhancement of the PHLPP-mediated Akt dephosphorylation, e.g. via FKBP51, could be an option to sensitize susceptible cancer cells to chemotherapy. However, to implement this strategy pharmacologically, a much better biochemical understanding of the Akt-FKBP51-PHLPP interaction is required. The aim of our study was thus to get an improved insight into the interaction of FKBP51 and Akt.

## Results

### Numerous FKBPs can Bind Directly to Akt

**Figure 1:**
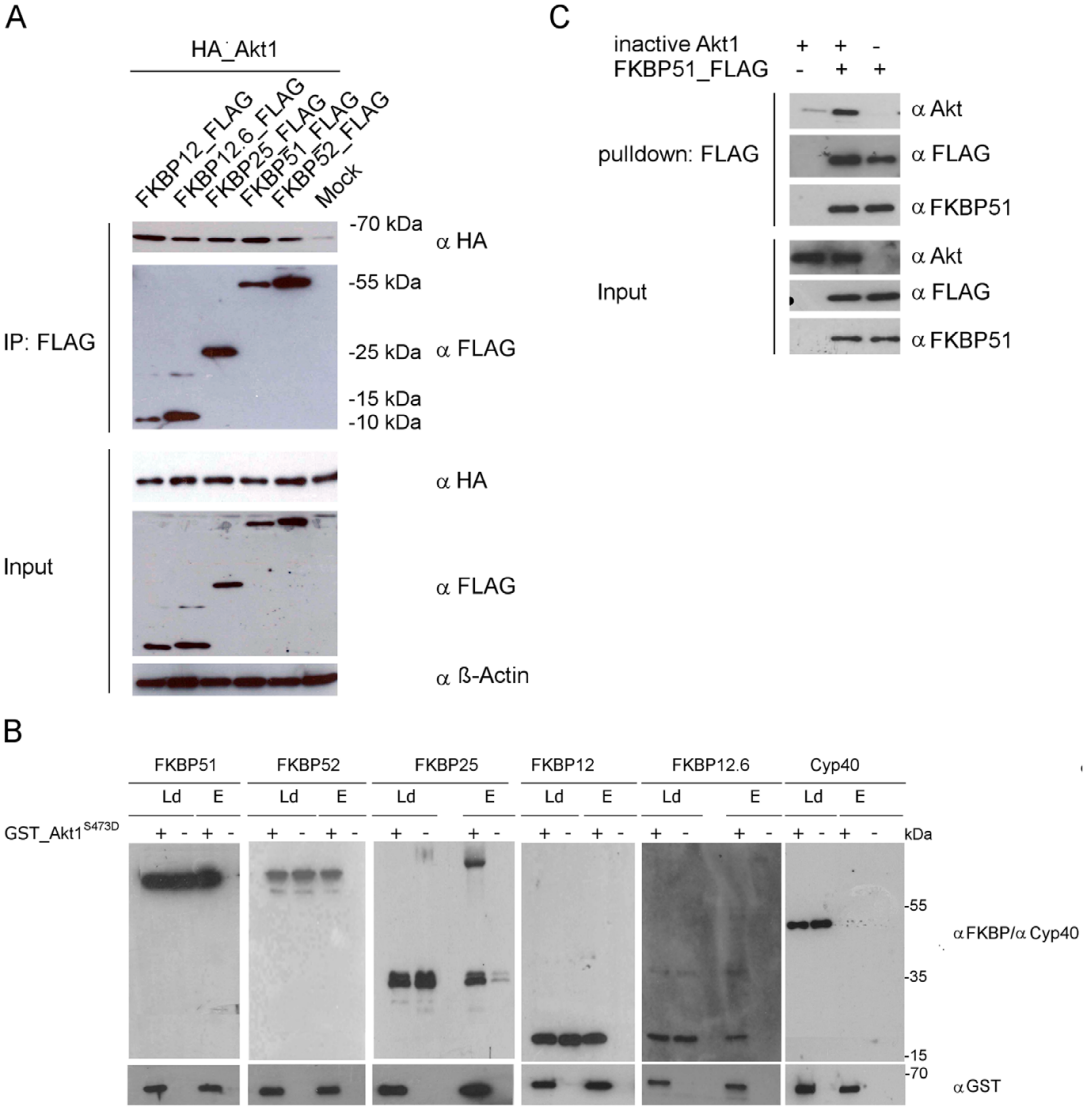
Akt1 binds to several FKBPs and this interaction is direct. **A** HEK293T cells were transfected with HA_Akt1 and the indicated FKBP constructs. After 2 days the lysates were immunoprecipitated (IP) and analyzed by Western blotting. **B** Purified FKBPs and purified GST-tagged Akt1S473D (mimicking an active conformation) were subjected to a GST-pulldown followed by Western blotting. Cyclophilin, which also harbors a TPR domain, but no FK domains, was used as a negative control. (Ld = Load, E = eluates). **C** Purified FLAG-tagged FKBP51 and purified inactive Akt were mixed, subjected to a FLAG pulldown and analyzed by Western blotting.

Since members of the FKBP family are highly homologous to each other we asked if other FKBPs are able to bind to Akt. Surprisingly, in HEK293T cell lysates Akt1 also co-immunoprecipitated with FKBP52, FKBP25 and even with the smaller FKBP12 and 12.6, which consist only of the FK506-binding domain (Figure 1A). To examine whether these interactions are direct, we used purified GST_Akt1^S473D^ [26], a GST-tagged constitutively active Akt mutant, as well as purified FKBP proteins [27] and performed pulldown assays (Figure. 1B). All FKBPs bound to Akt1^S473D^ -loaded beads but not to empty beads or beads loaded with GST alone (Figure S1). No interaction was observed with purified Cyp40 [28], a closely related immunophilin, which also has a TPR domain and binds to Hsp90 but which lacks an FK506-binding domain. The direct interaction with purified FKBP51 was confirmed in a reversed pulldown using inactive untagged Akt1. Again, Akt1 was pulled down in the presence, but not the absence, of FKBP51 (Figure 1C).

### FKBP51 can Bind to Multiple AGC Kinases

**Figure 2:**
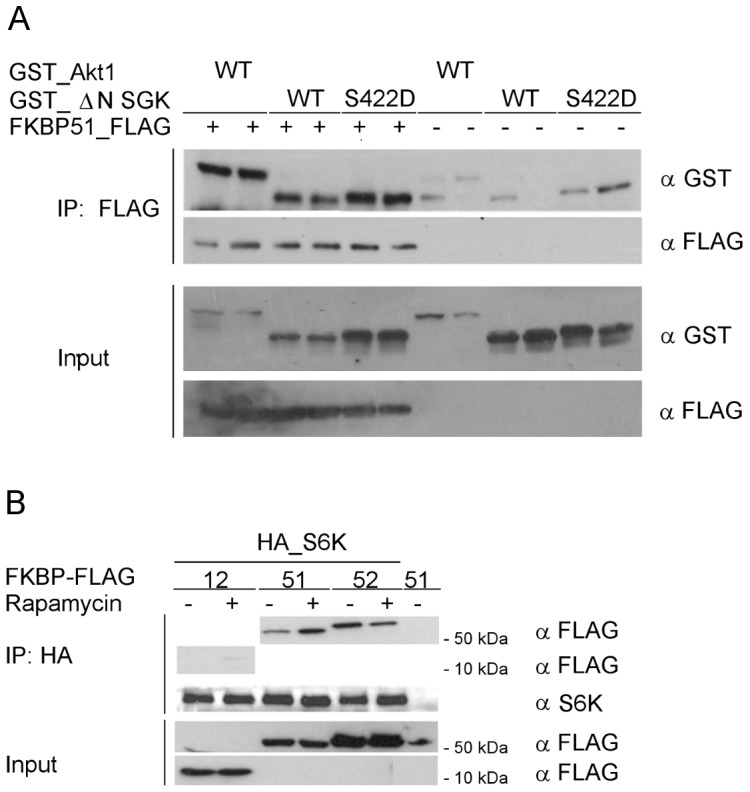
Other AGC kinases can also bind to FKBP51. **A** HEK293T cells were co-transfected in duplicates with GST-tagged Akt1 or ΔN_SGK^S422D^ and FLAG-tagged FKBP51. After 48 h, the lysates were immunoprecipitated and analyzed in duplicates by Western blotting. **B** HeLa cells were co-transfected with FLAG-tagged FKBPs and HA-tagged S6K, treated with rapamycin (25 nM) or DMSO for 60 min and lysed. Lysates were immunoprecipitated and analyzed by Western blotting.

It was shown that FKBP51 binds to Akt1 and Akt2 but not to Akt3 [23]. To test whether the interaction of FKBP51 is specific to Akt or whether other AGC kinases could also interact with FKBP51 we performed co-immunoprecipitation experiments with SGK and p70S6K. Both wildtype SGK and SGK harboring an activating S422D mutation, clearly co-immunoprecipitated with FKBP51 to a similar extent as GST-tagged Akt1 (Figure 2A). FKBP51 and FKBP52 co-immunoprecipitated also with p70S6K overexpressed in HeLa cells (Figure 2B) while FKBP12 only marginally bound to p70S6K.

### Influence of the PH Domain of Akt and its Phosphorylation Status on the Interaction with FKBP51

**Figure 3:**
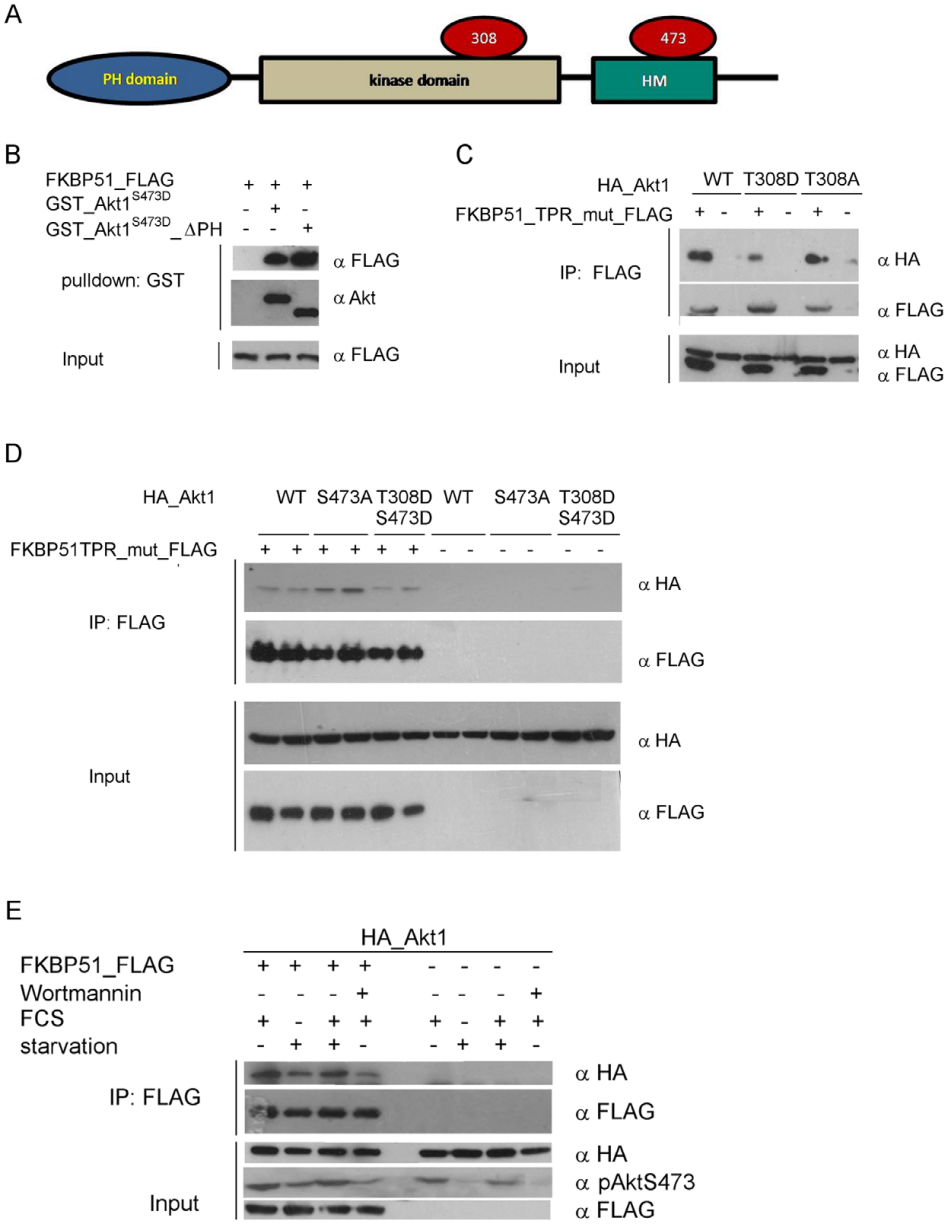
Influence of the PH domain and the activation status of Akt. **A** Domain structure of Akt **B** Purified GST-tagged active Akt or a mutant lacking the PH domain were incubated with purified FLAG-tagged FKBP51. After 3 h, the interaction was tested by GST pulldown and Western blotting. **C** and **D** HEK293T cells were transfected with the indicated HA-tagged Akt1 phosphorylation site mutants with or without co-transfected FLAG-tagged FKBP51^K352A/R356A^ (TPR_mut). After 2 days cells were collected, lysed, immunoprecipitated and analyzed by Western blotting (duplicates were analyzed for D). **E** HEK293T cells were starved for 16 h, stimulated with FCS for 45 min or treated with wortmannin. Controls were incubated in the presence of 10% FCS and treated with DMSO. Cells were lysed, immunoprecipitated and analyzed by Western blotting.

Next, we explored which domain of Akt is responsible for binding to FKBP51. Therefore, we performed pull-down assays with full length Akt and with an Akt construct lacking the PH domain (Figure 3A). Both constructs interacted identically with FKBP51 indicating that the PH domain is not necessary (Figure 3B). This is consistent with the observed interaction of FKBP51 with S6K and SGK, two kinases that lack the PH domain. The conformation and activity of Akt1 is regulated by phosphorylation at T308 and S473. To investigate the influence of these important sites we performed immunoprecipitation assays with HEK273T cell co-expressing FKBP51 (containing a TPR-mutation to exclude confounding influences of Hsp90) together with Akt1 containing a series of phosphorylation-resistant or phosphomimetic substitutions at T308 and/or S473. All these Akt constructs co-immunoprecipitated specifically with FKBP51 (TPR mutant) but not with mock-transfected controls (Figure 3C and Figure 3D). The phosphorylation status of T308 in the activation loop of Akt was not important for the interaction with FKBP51 under these cellular conditions whereas the phosphoresistant mutation S473A (lane 3 and 4) slightly increased binding of FKBP51. We next controlled the Akt activation status by stimulating or starving the cells or by inhibition of the PI3K pathway using wortmannin (Figure 3E). As expected, starvation (lane 2) and wortmannin treatment (lane 4) reduced phosphorylation of Akt at S473 and correlated with a slightly reduced binding to FKBP51. The underlying reasons for discrepancy to the results observed with the S473A mutant remain to be established. Contrary to the findings by Pei et al. [23], we observed an increase – not a reduction – in Akt S473 phosphorylation upon co-expression of FKBP51.

### Allosteric but not ATP-competitive Akt Inhibitors Diminish the Interaction with FKBP51

**Figure 4:**
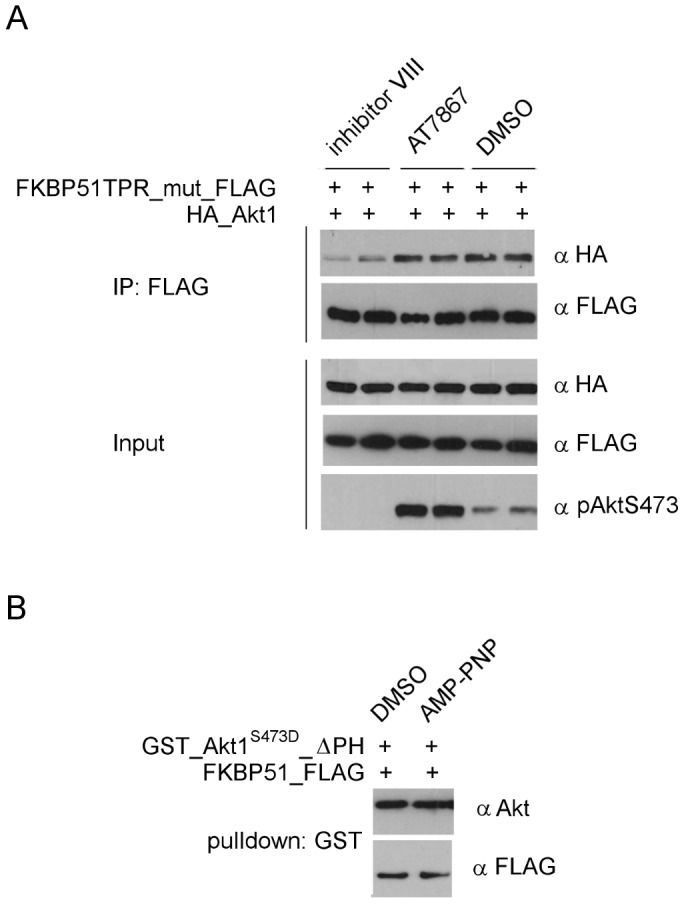
The FKBP51-Akt interaction depends on the conformation of Akt. **A** HEK293T cells were transfected with FLAG-tagged FKBP51^K352A/R356A^ (TPR_mut) and HA-tagged Akt1. After 2 days cells were treated with 10 µM inhibitor VIII, AT7867 or DMSO for 1 h. Cell lysates and immunoprecipitates were analyzed in duplicates by Western blotting. **B** GSH beads loaded with purified activated GST_Akt1ΔPH were incubated with FKBP51 with or without AMP-PNP. Eluates were analyzed by Western blotting.

Since Akt activation seemed to influence the interaction with FKBP51 at least to a certain degree we next sought to control the conformation of Akt more directly using Akt conformation-specific inhibitors. We used a classical ATP-competitive inhibitor (AT7867) (Figure 4A, lane 2), which binds and stabilizes the activated ‘PH out’ conformation of Akt (PDB code 2UW9) [29] by preventing access of phosphatases [30], and the allosteric inhibitor (inhibitor VIII) (lane1), which intercalates between the PH and the kinase domain of Akt and locks the latter in a closed inactive conformation (PDB code 3O96) [31]. As expected, the ATP-competitive inhibitor (AT7867) led to Akt hyperphosphorylation [30], [32] but it did not affect the interaction with FKBP51 (Figure 4A). This was confirmed *in vitro* by pulldown assays using the non-hydrolyzable ATP analog AMP-PNP (Figure 4B). As described, the allosteric inhibitor completely abolished cellular Akt S473 phosphorylation [30], [33]. Interestingly, this compound substantially reduced binding of Akt to FKBP51. This suggests that in the conformation stabilized by inhibitor VIII the binding site with FKBP51 might be masked.

### Multiple Domains of FKBP51 Contribute to the Binding to Akt

**Figure 5:**
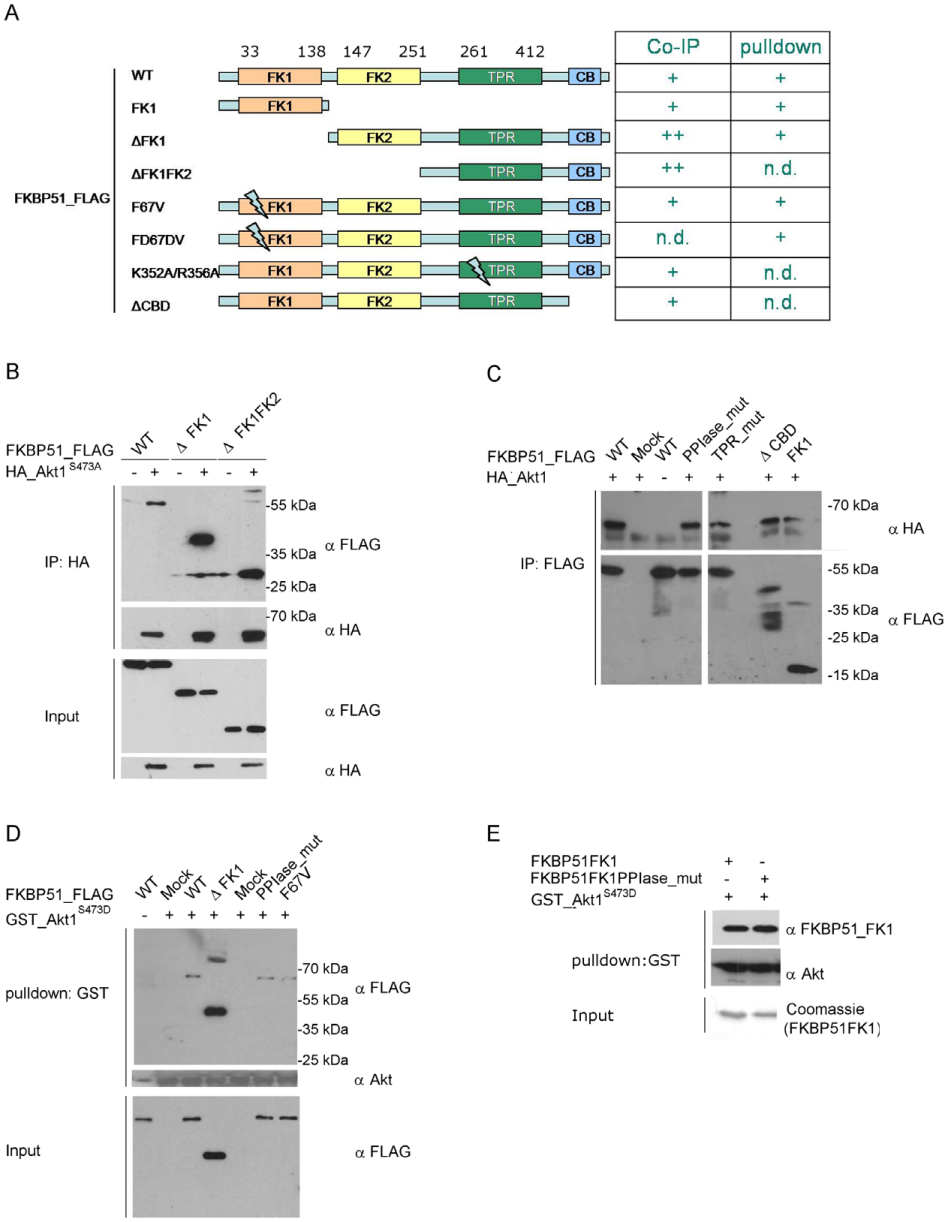
Mapping of the FKBP51 domains interacting with Akt. **A Left:** Overview of the constructs used to map the FKBP51/Akt interaction. **Right:** Summary of the Akt interactions of FKBP domains investigated in HEK293T cells by immunoprecipitation or by pulldown with purified proteins. (WT = wildtype). **B** and **C** HEK293T cells were transfected with the indicated constructs. After 48 h, lysates were prepared and HA- or FLAG-immunoprecipitation was performed, followed by Western blotting. **D** GSH beads loaded with purified GST-tagged Akt1^S473D^ were incubated with the indicated FLAG-tagged FKBP51 mutants for 3 h. After elution a Western blot analysis was performed. **E** GSH beads loaded with purified GST-tagged Akt1^S473D^ were incubated with the indicated FLAG-tagged FKBP51FK1 constructs for 3 h. After elution a Western blot analysis was performed and the detected with an antibody which detects the FK1 domain of FKBP51.

We next aimed to map the domains of FKBP51 that interact with Akt. First we truncated the FK506-binding domain (ΔFK1) and the FK1-like domain (ΔFK1FK2) (Figure 5A). Both deletion constructs co-immunoprecipitated with overexpressed Akt1 (Figure 5B). We also co-expressed Akt1 with two FKBP51 mutants where the PPIase activity of the FK1 domain or the Hsp90-binding capacity of the TPR domain was abolished. We also tested a construct lacking the putative C-terminal calmodulin- binding site and the isolated FK506-binding domain (FK1). In all cases, Akt1 co-immunoprecipitated with the FKBP51 constructs, although with slightly reduced efficiency for the mutants (Figure 5C). To confirm the capacity of multiple domains of FKBP51 to interact with Akt we performed pulldown assays using purified proteins (Figure 5D). The functionality of the FKBP51 proteins (with exception of the FKBP51 ΔFK1 construct) was verified by an active site titration for the FK506-binding pocket [27]. Again, all FKBP51 constructs were retained by Akt1 to a similar extent. The independence of the PPIase activity was further confirmed using a pulldown assay with the isolated FK506-binding domain of FKBP51 together with the corresponding PPIase-deficient mutant. Both proteins bound to Akt to a similar extent (Figure 5E).

### FKBP Inhibitors do not Disrupt FKBP-Akt Interaction

**Figure 6:**
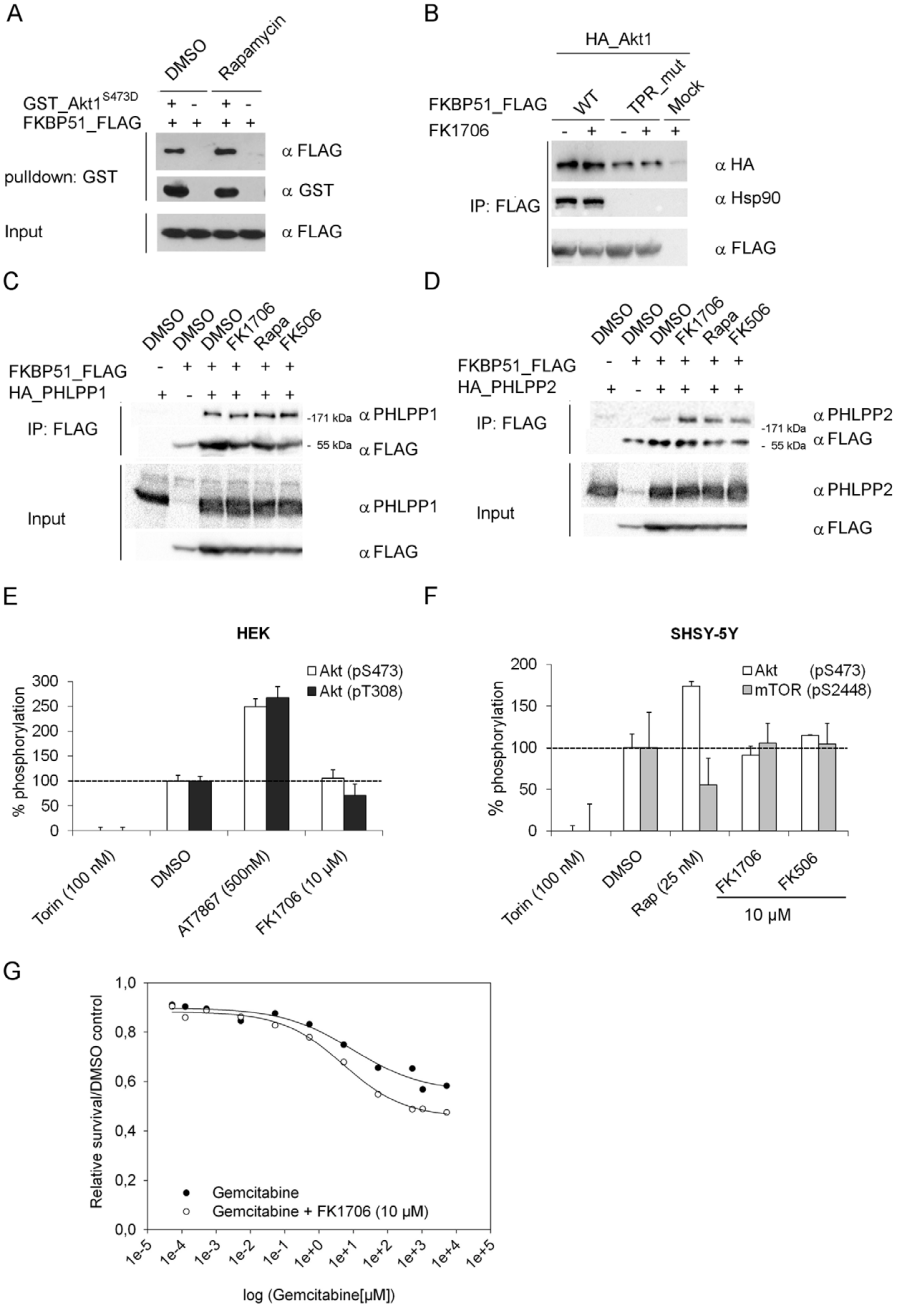
Influence of FKBP inhibitors. **A** Purified proteins were mixed and treated with DMSO or rapamycin (1 µM). After 3 h a GST-pull-down was performed followed by Western blotting. **B** HEK293T cells were transfected with the indicated constructs. After 48 h 1 µM FK1706 was added for 1 h. The lysates were immunoprecipitated and a Western blot was performed. **C and D** HEK293T cells were transfected with FLAG-FKBP51 and HA-tagged PHLPP1 or HA-tagged PHLPP2. After 48 hFKBP inhibitors (1 µM) or DMSO were added for 1h. Lysates were immunoprecipitated and analyzed by Western blotting. **E** HEK293T cells were stimulated with FCS for 1 h in the presence of the indicated compounds. After cell lysis, cellular Akt phosphorylation was determined using a homogeneous time- resolved FRET assay. **F** SHSY-5Y cells were stimulated with FCS for 1 h in the presence of the indicated compounds. After cell lysis, cellular Akt and mTOR phosphorylation was determined using a homogeneous time resolved FRET assay. **G** SU.86.86 cells were plated, treated with Gemcitabine in the absence or presence of FK1706. Cell survival relative to DMSO treated controls was determined.

The ability of several FKBP members to bind to Akt (Figure 1) suggested the FK506-binding pocket common to all these proteins as an interaction site. We therefore tested if FKBP ligands blocking the PPIase domain can reduce binding of Akt to FKBP51. We first performed a pull-down experiment using purified FKBP51 and purified AktS473D as bait in the absence and presence of the high-affinity ligand rapamycin. The amount of FKBP51 that was specifically retained by Akt was not affected by an excess of rapamycin (Figure 6A). We next co-immunoprecipitated Akt with FKBP51 or its TPR-mutant in the presence or absence of the non-immunosuppressive FK506 analog FK1706 [34]. Binding of Akt was slightly reduced for the TPR-mutant but it was still significantly retained compared to background (Figure 6B). The interaction with neither FKBP51 construct was affected by the treatment with FK1706. Similar results were obtained in cells treated with FK506 or rapamycin (Figure S2). Since PHLPP is regulating Akt phosphorylation and is proposed to be part of the Akt-FKBP51-PHLPP complex [23] we explored whether FKBP inhibitors affected the FKBP51-PHLPP complex. FKBP inhibitors had no effect on the integrity of the complex of FKBP51 with PHLPP1 or PHLPP2 (Figure 6C, Figure 6D). Finally, we tested whether cellular Akt or mTOR phosphorylation would be affected by FKBP inhibitors. Neither the phosphorylation of Akt at T308 nor S473 was affected in HEK293T cells treated with high concentrations of FK1706. Under the same conditions the mTOR inhibitor Torin-1 reduced Akt phosphorylation at both sites [35], while the ATP-competitive inhibitor AT7867 enhanced it demonstrating that the assay was able to detect the dynamic regulation of Akt in these cells (Figure 6E). Similar results were obtained for Akt S473 and mTOR S2448 phosphorylation in FK1706 or FK506-treated SHSY-5Y (Figure 6F) and HeLa cells (Figure S3). Rapamycin which served as control stimulated and inhibited both phosphorylations in the expected way. Since FKBP51 was shown to regulate the sensitivity of pancreatic cancer cells to chemotherapeutics [23], [24] we tested the effect of FKBP inhibitors in these cells. In a cell viability assay we observed that FK1706 (10 µM) did not enhance the cytotoxic effect of Gemcitabine in SU.86.86 cells (Figure 6G).

## Discussion

The kinase Akt is a key signaling node which is essential for many adaptive processes (e.g., neuroregeneration [36], but detrimental when overactive like in many cancers. In turn, the activity of Akt is tightly regulated under normal conditions. Recently, FKBP51 emerged as a new regulator of Akt. FKBP51 is a Hsp90-cochaperone and a target of the clinically used immunosuppressants or anticancer-drugs FK506 and rapamycin [37]. While the role of protein kinases in cancer development is well established, the influence of FKBP51 on malignancy is contradictory. Several studies have shown that FKBP51 is up-regulated in several cancers and that it can promote the proliferation of many cancer cell lines [25], [38]-[41]. In contrast, Wang and colleagues found FKBP51 to enhance the response to chemotherapeutics, such as Gemcitabine in pancreatic and breast cancer cell lines [23], [24]. Mechanistically, the latter effect was attributed to a scaffolding function of FKBP51, which could promote dephosphorylation of Akt by the phosphatase PHLPP. However, the exact interaction mode of FKBP51 with AKT and PHLLP is not known.

**Figure 7:**
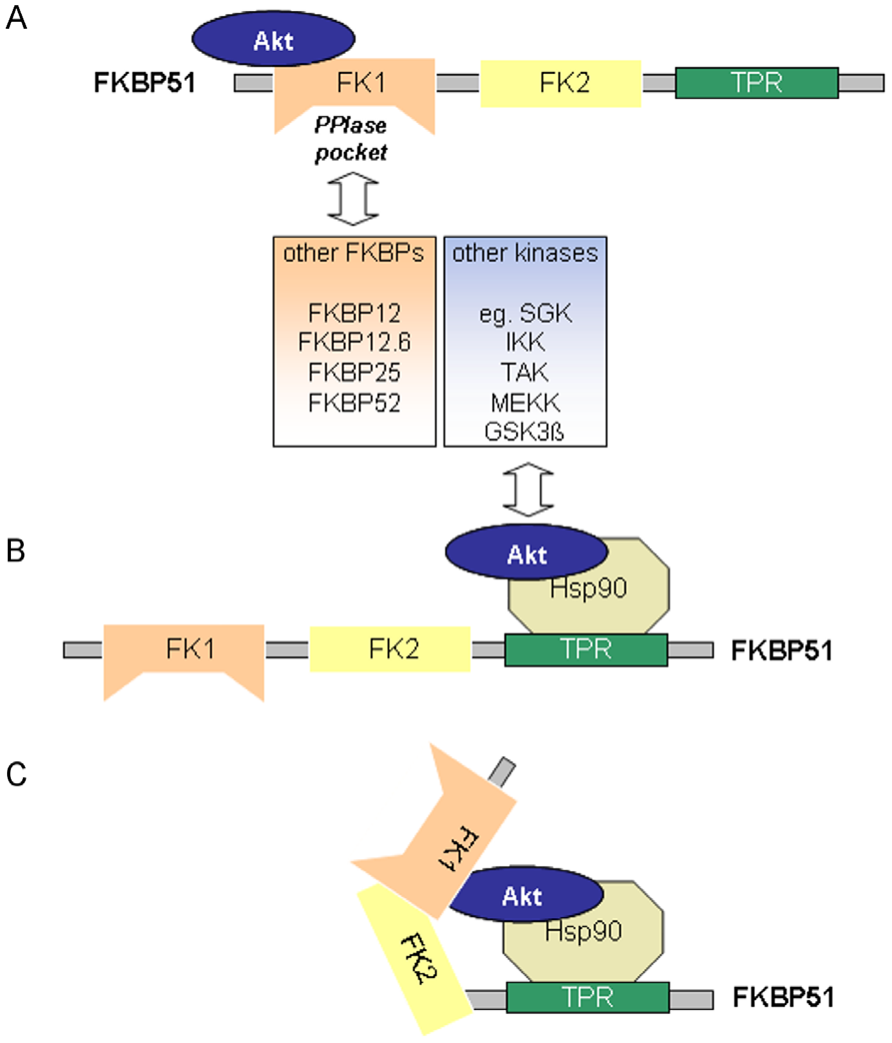
Schematic model of possible Hsp90-Akt-FKBP51 complexes. **A** FKBP51 can bind directly to Akt via its FK1 domain, but not with its FK506-binding pocket. Several other FK1-possessing FKBP homologs may bind to Akt in a similar mode. **B** Akt and several other kinases can bind to FKBP51 indirectly via Hsp90. **C** FKBP51 could assist the chaperoning of Akt by binding to Hsp90 via its TPR domain and by interacting with Akt via its FK1 domain.

Our study supports a robust FKBP51-Akt1 interaction but we were unable to observe enhanced dephosphorylation upon FKBP51 expression. This could be due to PHLPP not being the rate-limiting phosphatase for Akt in our cells and under our cell culture conditions. According to our results the interaction of Akt and FKBP51 might be more complex than previously thought (Figure 7).

First, the interaction is not restricted to FKBP51 since Akt can bind to several FKBPs. Whether different FKBPs can compete for a similar binding site on Akt and whether this could be important for the effect of individual FKBPs on Akt remains to be established. For example, other FKBPs could displace FKBP51 from the Akt-PHLPP complex in a way reminiscent of the opposing effects of FKBP51 and FKBP52 on steroid hormone receptors [20] (Figure 7A).

Second, FKBP51 can interact with several AGC kinases in addition to Akt. Similarly, kinases from other classes have previously been reported to bind to FKBP51 [30], [42], [43]. The signaling of Akt, SGK and S6K is highly interconnected. Any effects observed on the PI3K-Akt-mTOR pathway after FKBP51 overexpression or downregulation are thus not necessarily being mediated via Akt but could be due to modulation of any of those kinases. Whether the binding to SGK or S6K is direct or via a third partner (e.g., Hsp90) is currently unclear (Figure 7B).

The PH domain itself is not required for the FKBP51-Akt interaction and is absent in other protein kinases that are also interaction partners of FKBP51. The best indication where FKBP51 binds on the Akt surface was obtained using the conformation-specific Akt inhibitors. The structures of Akt in complex with AT7867 and inhibitor VIII show that most of the core C- and N-lobes are structurally conserved, indicating that most regions of the conserved kinase domain may not provide the key interaction sites with FKBP51. The most prominent difference in the conformations of Akt stabilized by AT7867 and by inhibitor VIII is the rearrangement of the αC-helix (residues 185–205), which is stabilized in the presence of AT7867 allowing the binding of the HM to the PIF-pocket and destabilized in complex with inhibitor VIII. In addition, the activation loop (residues 292–313) is completely occluded by the PH domain in the presence of inhibitor VIII. Interestingly, the attachment of the PH domain to the catalytic domain of Akt occluding the activation loop, as observed in complex with inhibitor VIII, is thought to occur in the inactive conformation of Akt [44], [45], to which FKBP51 also binds. Therefore, a key binding site for FKBP51 is unlikely to lie within the PH-domain interaction site on the catalytic domain. Rather, the interaction site may exist at or within the proximity of the actual site where inhibitor VIII binds on the catalytic domain or at allosteric sites affected by the interaction with inhibitor VIII. Interestingly, the binding of inhibitor VIII to Akt completely disrupts the formation of the αC-helix highlighting this region, which appears highly flexible in AGC kinases in solution, as the potential common recognition site for kinases by FKBPs.

Third, the Akt-FKBP51 interaction is probably bimodal at the biochemical level (Figure 7C). Binding of Akt to FKBP51 is mediated in part by Hsp90 since it is partially affected by Hsp90-disrupting mutations. However, FKBP51 can clearly bind to Akt also directly via the FK1 domain. This is consistent with the domain mapping of FKBP51 where all constructs that contained either a functional TPR (Figure 5B–D) domain or the FK1 domain (Figure 5E) were able to bind to Akt. The only exception is the pull-down of purified FKBP51 Δ FK1_FLAG, where FKBP51 lacks the FK1 domain and cannot bind via Hsp90 since the latter is lacking in the purified reconstituted system. This may indicate that FKBP51 can bind to Akt also via the FK2 domain or it could be due to misfolding of this construct and spurious binding of Akt (ΔFKBP51 FK1_FLAG was the only construct that could not be checked by active site titration prior to the pull-down assays). The two-domain interaction model also raises the possibility that FKBP51 might use both interaction sites to regulate Akt within a FKBP51-Hsp90-Akt complex, similar to the putative regulation of steroid hormone receptors by FKBP51.

All FKBPs contain a highly conserved FK506 binding site, which displays the PPIase activity but which can also mediate protein-protein interactions [46]. The finding that all FKBPs, but not Cyp40, bound to Akt strongly suggested the common FK506-binding site as the connector to Akt. However, binding of FKBP51 to Akt was not affected by several high-affinity ligands, neither in purified systems nor in cells were the alternative binding mode via Hsp90 was controlled for. Likewise, mutations in the FK506-binding site, which abolish the PPIase activity, did not affect binding to Akt. This suggests that other parts of the FK1 domain of FKBP51 interacted with Akt. While other parts of the FK506-binding domain are less conserved between the different FKBP homologs they still share a highly conserved structural fold [47], which could be important for binding to Akt.

The inability of FKBP51 ligands to disrupt the FKBP51-Akt interaction suggests that the clinically used FKBP ligands are unlikely to affect the regulation of Akt by FKBP51. This is consistent with the lack of an effect of the high-affinity ligands FK506 or FK1706 on the Akt-mTOR pathway in several studied cell types. Likewise, the sensitivity towards cytostatic agents, which was reported to be suppressed by FKBP51 [23],[24], was not affected by FK1706. At the biochemical level, however, parts of the FK1 domain, which must be in the vicinity of the FK506-binding site, seem to be important for the interaction with Akt. This raises the possibility to develop ligands for the FK506-binding site that might be able to allosterically modulate the FKBP51-Akt interaction. The feasibility of this hypothesis will require a better understanding of the parts of FKBP51 that bind to Akt.

## Materials and Methods

### Plasmids and other Materials

The pcDNA3 constructs for expression in mammalian cells were generated using the primer pairs 5′-CGGAATTCATGGACTACAAGGACGATGACGATAAGATGGGAGTGCAGGTGGAAACCATC-3′ and 5′-GG CTCGAGTCATTCCAGTTTTAGAAGCTCCACA-3′ (FKBP12_FLAG) 5′CGGAATTCATGGACTAC AAGGACGATGACGATAAGATGGGCGTGGAGATCGAGACCATC-3′ and 5′-GGCTCGAGTCACTCTAAGTTGAGCAGCTCC-3′ (FKBP12.6_FLAG) 5′-CGGAATTCATGGACTAC AAGGACGATGACGATAAGATGGCGGCGGCCGTTCCACAGCGG-3′ and 5′-GG CTCGAG TCAATCAATATCCACTAATTCCACTT-3′ (FKBP25_FLAG), 5′-CGGAATTCATGACTACTGATGAAGGTGC-3′ and 5′-GCAGTCGACTCTCCTTTGAAATCAAGGAGC –3′ (FKBP51_FLAG) 5′-CCGAATTCATGACAGCCGAGGAGATG-3′ and 5′-GTCGACTCATTCTCCCTTAAACTCAAACAACTC-3′ (FKBP52_FLAG) 5′-ACCAGGTACCATGTTTGAAGATGGAGGCATTATCCG-3′ and 5′-TTTTTTGAATTCTCACTTGTCATCGTCGTCCTTGTAGTC-3′ (FKBP51ΔFK1_FLAG), 5′-CCGAGGTACCATGGATACCAAAGAAAAATTGGAGCAG-3′ 5′-TTTTTTGAATTCTCACTTGTCATCGTCGTCCTTGTAGTC-3′ (FKBP51ΔFK1FK2_FLAG).

The amplified cDNA was cloned into the pcDNA3 vector using the restriction sites EcoRI and Xho, XbaI and KpnI or KpNI and EcoRI. pRK5 constructs were a kind gift of Dr. Theo Rein (MPI for Psychiatry, Munich, Germany) [48].

His-tagged full length FKBPs and Cyp40 were expressed as described [27], [28]. pProExHta_ΔFK1 was generated using the primers 5′-GGGAGAATTCTTTGAAGATGGAGGCATTATCCG-3′ and 5′-TACCTCATACGTGGCCCTCAGGTTTCTC-3′. pProEx-Hta_FK1 was generated using the primers 5′- CATGCCATGGCAATGACTACTGATG-3′ and 5′-CAGTCGACTCACTCTCCTTTGAAATCAAGGAGC-3′. The constructs were cloned into pProExHta at the restriction sites EcoRI/KpNI and NcoI/SalI, respectively. Point mutations were obtained with the QuikChangeII*®* Site-Directed Mutagenesis Kit (Agilent Technologies, Santa Clara, CA).

pCMV5.HA_Akt1WT, pCMV5.HA_Akt1^S473A^, pCMV5.HA_Akt1T^308D/S473D^ plasmids were a kind gift of Dr. Brian Hemmings and Dr. Peter Cron (FMI, Basel, Switzerland). pEBG2T-Akt1ΔPH, pEBG2T-ΔNSGK and pEBG2T-ΔNSGK^S422D^ were desribed before [26]. pcDNA3-HA-PHLPP1 (Plasmid #22404), pcDNA3-HA-PHLPP2(Plasmid #22403) and pRK7-HA-S6K1 were purchased from Addgene (Plasmid #8984). Purified inactive Akt1 as well as wortmannin and inhibitor VIII were purchased from Merck Millipore, Billerica, MA.

All cell culture media (DMEM, D-PBS, foetal bovine serum (FBS), penicillin/streptomycin) were purchased from Gibco-Invitrogen (Carlsbad, CA). Rapamycin was bought from Cfm Oscar Tropitzsch (Marktredwitz, Germany) and FK1706 was a kind gift from Astellas Pharma Inc. (Tokyo, Japan). Inhibitor AT7867 was acquired from Selleckchem (Houston, TX). FK506, AMP-PNP and protease inhibitors were purchased from Sigma-Aldrich (Steinheim, Germany).

The antibodies against FKBP12, FKB12.6 and FKBP25 and ß-Actin were obtained from Abcam (Cambridge, UK). Antibodies against AktpS473 and Akt (pan) were purchased from Cell Signaling (Danvers, MA). Antibodies against FKBP51 and FKBP52 were purchased from Bethyl Laboratories (Montgomery, AL). Antibodies against Cyp40 and Hsp90 were provided by Santa Cruz Biotechnology (Santa Cruz, CA). The antibody against the FK1 domain (FFI) was a kind gift of M. Cox [49]. Nickel-NTA agarose was from Qiagen (Hilden, Germany).

### GST Pulldown Assay

15 µM FKBPs or Cyp40 were combined with 22 µM GST_Akt1^S473D^ or GST_Akt1^S473D^_ΔPH coupled to 10 µl glutathion resin (Miltenyi Biotech, Bergisch Gladbach, Germany) and incubated for 3 h in 500 µl NETN buffer (20 mM Tris-HCl [pH 8.0], 100 mM NaCl, 1 mM EDTA, 0.5% igepal, 50 mM glycerophosphate and 10 mM NaF, 0,5 mM natriumdihydrogenorthovanadat) at 4°C. AMP-PNP (5 mM), rapamycin (1 µM) or DMSO were added where indicated. After 3 h the resin was collected, washed 5 times with each 1 ml NETN buffer and finally boiled in 20 µl SDS sample buffer. The supernatant was subjected to SDS gel electrophoresis and immunoblotting.

### FLAG Pulldown Assay

10 µl FLAG column saturated with FKBP51_FLAG was incubated with 20 µM inactive Akt (Millipore) in 500 µl NETN buffer at 4°C overnight. The next day the resin was collected, washed 5 times with NETN buffer and eluted with FLAG peptide according to the manufacturer’s instruction. The elution was subjected to SDS-PAGE and immunoblotting.

### Cell Culture and Transfection

HEK-293T cells were maintained in DMEM, supplemented with 10% FBS and 1% antibiotics (penicillin, streptomycin) solution. For transfection, cells were plated in 6-well plates and subjected to Lipofectamine 2000 transfection procedure as recommended by the manufacturer (Invitrogen). Compounds were added where indicated to the culture medium (Inhibitor VIII or AT7867, each 10 µM, 1 h), FK506, rapamycin or FK1706 (each 1 µM, 1 h), wortmannin (200 nM, 6 h). Cells were starved in FCS free medium for 16 h and stimulated with FCS (30 min) when indicated.

### Cell Lysis and Immunoprecipitation

After stimulation and/or treatment with the indicated compounds cells were washed twice with PBS and lysed in NETN buffer containing 1% (v/v) protease inhibitor mix (Sigma-Aldrich) and PMSF (1 mM) (Carl Roth, Karlsruhe, Germany) by shaking at 1000 rpm at 4°C. After 20 min the supernatant was collected by centrifugation 4°C at 14.000 rpm. Total protein was determined by BCA Assay (Thermo Fisher Scientific, Rockford, IL). Cell lysates were combined with FLAG or HA affinity resin and incubated under moderate agitation overnight at 4°C. Beads were pelleted or collected with the magnet and washed 5 times with lysis buffer. Finally, beads were directly boiled after addition of an equal amount of SDS sample buffer for 5 min and SDS-Page followed by immunoblotting was performed.

### *In vivo* Phosphorylation Assays for Akt and mTOR

Phosphorylation at Akt(S473), Akt(T308) and mTOR(S2448) was assessed using a homogeneous time-resolved fluorescence assay (Cisbio, Codolet, France) according to the manufacturer’s recommendations. Briefly, HEK293, SH-SY5Y or HeLa cells were grown in 96-well plates in FCS in the presence of 10 µM FKBP ligand or 100 nM torin-1 for 60 min. Cells were lysed and the lysates were transferred to a white 384-well plate. After addition of d2-labeled anti-Akt or anti-mTOR antibody and K-labeled anti-phospho-Akt or anti-phospho mTOR antibody, the mixtures were incubated at room temperature for 4 h or overnight. FRET efficiency was determined by the emission ratios 665 nm/620 nm after excitation at 320 nm using a Tecan GENios Pro reader. The data were normalized to the results for Torin-1 (0%) and DMSO (100%).

### Cell Viability Assay

Cell viability of SU.86.86 cells treated with gemcitabine or gemcitabine with additional FK1706 was assessed using the CellTiter-Fluor Cell Viability assay from Promega, Madison, WI according to the manufacturer’s protocol. Briefly, cells were plated into a 96-well plate and treated with gemcitabine (0,126 nM-5 mM) or gemcitabine+FK1706 (10 µM) for 72 h. Thereafter, CellTiter-Fluor reagent was added and cells were incubated at 37°C for 30 min. Fluorescence was measured at an excitation of 370 nm and an emission of 520 nm using a Tecan GENios Pro reader. DMSO treated cells served as control and were correlated with treated cells.

## Supporting Information

Figure S1 **HEK293T transfected with the indicated constructs were treated with or without 1 µM FK506 or rapamycin 1 µM for 1 h followed by immunoprecipitation and western blotting.**
(TIFF)

Figure S2 **GST protein and GST_Akt^S473D^ΔPH were coupled to GSH beads. Beads were incubated for 3 h with FKBP51_FLAG and eluted with GSH followed by western blotting.**
(TIFF)

Figure S3 **HeLa cells were stimulated with FCS for 1 h in the presence of the indicated compounds.** After cell lysis, cellular Akt and mTOR phosphorylation was determined using a homogeneous time- resolved FRET assay.
(TIFF)
