# Supplementary figures and images for: InterAKTions with FKBPs - Mutational and Pharmacological Exploration
Source: PLoS One. 2013 Feb 28;8(2):e57508. doi: 10.1371/journal.pone.0057508 (PMC3585324; doi:10.1371/journal.pone.0057508)

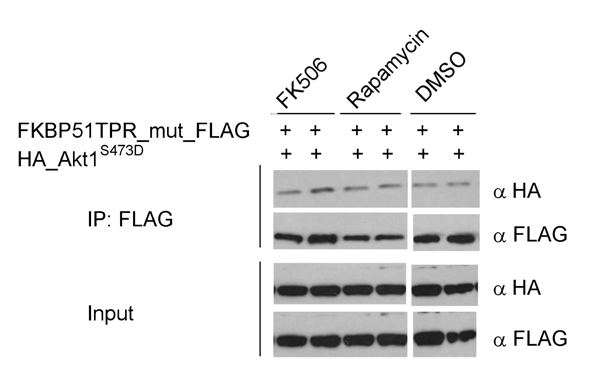

Supplement: Figure S2 — GST protein and GST_AktS473DΔPH were coupled to GSH beads. Beads were incubated for 3 h with FKBP51_FLAG and eluted with GSH followed by western blotting. (TIFF) [file pone.0057508.s002.tiff]

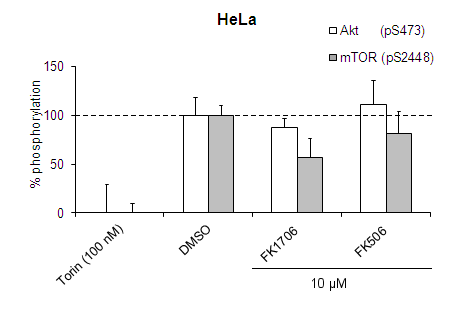

Supplement: Figure S3 — HeLa cells were stimulated with FCS for 1 h in the presence of the indicated compounds. After cell lysis, cellular Akt and mTOR phosphorylation was determined using a homogeneous time- resolved FRET assay. (TIFF) [file pone.0057508.s003.tiff]
